# Clinical Study on Combining Traditional Chinese Medicine With Acupuncture for Treating Insomnia Accompanied by Anxiety

**DOI:** 10.1002/brb3.71431

**Published:** 2026-06-12

**Authors:** Xue‐Wen Mao, Peng Bai

**Affiliations:** ^1^ Shunyi Hospital Beijing Traditional Chinese Medicine Hospital Beijing China; ^2^ Beijing University of Chinese Medicine Beijing China; ^3^ School of Acupuncture‐Moxibustion and Tuina Beijing University of Chinese Medicine Beijing China

**Keywords:** acupuncture treatment, anxiety, insomnia, Qi stagnation constitution, traditional Chinese medicine

## Abstract

**Objective:**

This study aims to investigate the clinical efficacy of combining traditional Chinese medicine (TCM) with acupuncture in treating insomnia accompanied by anxiety.

**Methods:**

Utilizing a simple sampling technique, 120 patients diagnosed with insomnia accompanied by anxiety from July 1, 2022, to June 30, 2024, were collected and divided into three groups according to enrollment sequence: the acupuncture and TCM group (treated with TCM combined with acupuncture), the TCM group (treated solely with TCM), and the control group (treated with conventional Western medicine), each comprising 40 patients. Indicators such as sleep quality, anxiety, and depression levels were collected for comparative analysis.

**Results:**

Post‐treatment, all three groups exhibited improvements in sleep quality, sleep onset time, sleep duration, sleep efficiency, sleep disturbances, daytime dysfunction, and overall scores compared with before treatment (*p* < 0.05). Statistically significant differences were observed among the three groups in these indicators post‐treatment (*p* < 0.05). The different treatment methods similarly impacted the Hamilton Anxiety Scale (HAMA) and Hamilton Depression Scale (HAMD) scores across the three groups (*p* > 0.05). Further analysis against baseline scores revealed that the HAMA and HAMD scores at 4 and 8 weeks post‐treatment were lower than those at baseline (*p* < 0.05). A significant difference was found in the body constitution scores among the three groups post‐treatment, with the scores ranking from highest to lowest as follows: control group > TCM group > acupuncture and TCM group. The effectiveness rates among the three groups did not significantly differ (*p* = 0.210).

**Conclusion:**

The efficacy of treating Qi stagnation type insomnia with a combination of TCM and acupuncture is validated, offering significant relief from clinical symptoms of insomnia and warranting its recommendation for widespread clinical use.

## Graphical abstract

In treating Qi stagnation type insomnia, the combination of traditional Chinese medicine and acupuncture can improve sleep quality, alleviate anxiety and depression, and improve physical fitness. Compared to using traditional Chinese medicine decoction alone for treatment, the combination of traditional Chinese medicine decoction and acupuncture has higher clinical efficacy.

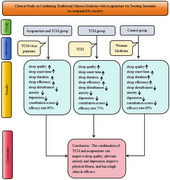

## 1 Introduction

Insomnia, a prevalent condition in today's society, substantially impairs the occupational, daily, and social functionalities of affected individuals, with a notable trend toward a younger demographic (Lauderdale et al. 2006). Defined by enduring dissatisfaction with sleep quality for a duration exceeding 1 month, the clinical manifestations of insomnia primarily include difficulty initiating sleep, early awakenings, difficulty maintaining sleep, and dreaminess, excluding secondary insomnia induced by psychological disorders, physiological illnesses, alcohol, or medication (Nishikawa et al. 2021). The incidence rate of insomnia among the adult population varies internationally from 30% to 50% (Fortier‐Brochu and Morin 2014), with a notable 45.4% prevalence reported within the Chinese demographic (Yin et al. 2017). A complex bidirectional relationship exists between insomnia and adverse emotional states such as depression and anxiety (Buysse et al. 2008). Prior research indicates that persistent insomnia or suboptimal sleep quality can disrupt the equilibrium between the sympathetic and parasympathetic nervous systems, leading to anxiety, depression, and additional negative emotions (Benca and Peterson 2008).

Insomnia frequently co‐occurs with a range of symptoms, with up to 40% of individuals suffering from insomnia also developing additional mental health disorders, predominantly anxiety and depression. In clinical settings, insomnia accompanied by anxiety is particularly prevalent, and the rapid economic development and accelerated social pace contribute to an increased likelihood of its occurrence (Shan et al. 2024). Currently, the clinical management for patients with co‐occurring insomnia and anxiety includes pharmacological treatments, psychological interventions, physical therapies, and specialized approaches such as TCM and acupuncture (C. Wang et al. 2021; G. T. Xue 2016). Despite the predominance of oral Western medications in treating this condition, the adverse and addictive properties of such medications have led to a growing acceptance of TCM and acupuncture among patients.

The TCM approach to insomnia treatment is extensively documented, with foundational principles such as “supplementing where there is deficiency and reducing where there is excess, balancing deficiency and excess” articulated as early as in the “Ling Shu” (The Spiritual Pivot), a theory that remains applicable to the present day. The primary pathogenesis of insomnia involves an imbalance where excess Yang fails to integrate with Yin. Historically, medical practitioners have offered diverse perspectives on insomnia treatment. In contemporary life, the ubiquitous increase in stress and the accumulation of negative emotions can readily culminate in a Qi stagnation constitution, with insomnia being particularly susceptible to emotional influences. Studies have demonstrated a clear correlation between insomnia and a Qi stagnation constitution, with a high proportion of Qi stagnation constitution among TCM constitutional types in insomnia (S. Li, Zhu, et al. 2023). Qi stagnation is the most common body constitution among insomnia patients (21%–44%) (L. Xue et al. 2020). Further studies suggest that individuals with higher Qi stagnation scores are more prone to early awakenings and difficulties initiating sleep (X. L. Jiang and Zhang 2012). Evidence shows that Qi deficiency and Qi stagnation constitutions were associated with depression, whereas the Qi stagnation constitution was associated with anxiety in systemic sclerosis patients (Kong et al. 2023).

Consequently, treatment strategies should not solely focus on insomnia's symptomatic manifestations but also aim to address its root cause, namely the Qi stagnation constitution.

Constitutional regulation primarily involves TCM‐specific formula interventions and acupuncture point conditioning. Although numerous studies have investigated the application of TCM and acupuncture for insomnia treatment, there is a scarcity of research on the combined application of acupuncture and TCM for constitutional regulation and subsequent insomnia treatment. Therefore, this study aims to conduct a clinical observation on the efficacy of treating Qi stagnation constitution insomnia with a modified prescription based on Chaihu Shugan Powder combined with acupuncture, drawing from clinical experience with constitution as the entry point and combining relevant TCM literature research, to broaden the perspectives and methods for clinical treatment of insomnia.

## 2 Study Subjects and Methods

### 2.1 Study Subjects

**FIGURE 1:**
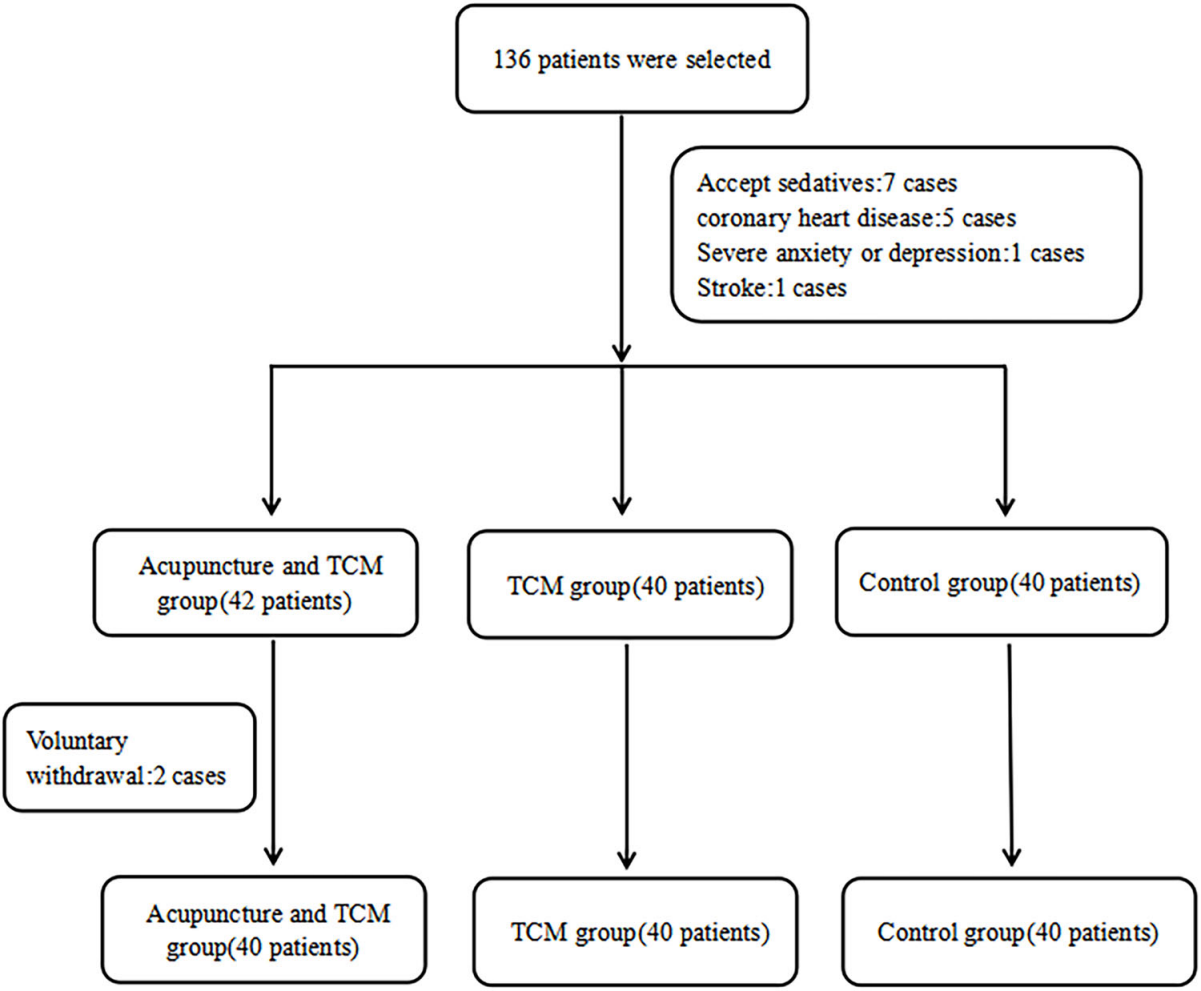
The process of participant screening.

Utilizing a simple sampling technique, 120 patients diagnosed with insomnia accompanied by anxiety from July 1, 2022, to June 30, 2024, were collected and divided into three groups according to enrollment sequence: the acupuncture and TCM group (treated with TCM combined with acupuncture), the TCM group (treated solely with TCM), and the control group (treated with conventional Western medicine), each comprising 40 patients. The process of participant screening is shown in Figure 1.

The diagnostic criteria for Western medicine were derived from the “Guidelines for the diagnosis and treatment of insomnia in China” (Chinese Sleep Research Society 2017), outlining insomnia as follows: (1) primary clinical symptoms of sleep disorder, including challenges in initiating sleep, dreaminess, light sleep, and in severe cases, total inability to sleep throughout the night, possibly accompanied by early awakening or difficulty resuming sleep, daytime fatigue, and listlessness; (2) the sleep disorder occurs ≥ 3 times weekly and persists for ≥ 3 months; (3) insomnia results in emotional instability or psychiatric symptoms, reduced activity efficiency, or impairs social functionality; and (4) not related to any physical or psychiatric conditions.

TCM diagnostic criteria: Adherence to the diagnostic guidelines for “insomnia” as outlined in the “Thirteenth Five‐Year Plan” textbook “Internal Medicine of Traditional Chinese Medicine (New Century Fourth Edition)” (B. L. Zhang and Wu 2017). (1) For mild symptoms, difficulties in falling asleep or frequent awakenings, with reinitiating sleep being challenging for over three consecutive weeks; for more severe symptoms, complete inability to fall asleep throughout the night; (2) frequently accompanied by dizziness, palpitations, forgetfulness, headaches, numerous dreams, restlessness, and fatigue; and (3) commonly associated with abnormal emotions, disinterest in food and drink, lethargy, excessive anxiety, and post‐illness weakness.

Qi stagnation constitution diagnostic criteria: Evaluation is based on the “Classification and Determination of Constitution in TCM” issued by the Chinese Association of Traditional Chinese Medicine in 2009 (Chinese Association of Traditional Chinese Medicine 2009), with professionals calculating both raw and conversion scores from respondents’ responses. Raw score = total of item scores, conversion score = [(raw score—number of items)/(number of items × 4) × 100. Criteria for determination: A Qi stagnation constitution is identified with a conversion score of ≥ 40 points, excluding the consideration of mixed constitutions.

The inclusion criteria were as follows: (1) aged 18–65; (2) meeting the diagnostic criteria specified; (3) Pittsburgh Sleep Quality Index (PSQI) score ≥ 7; Hamilton Anxiety Scale (HAMA) score > 7 and ≤ 28; Hamilton Depression Scale (HAMD) score < 7; and (4) no treatment with sedative or anti‐anxiety medications in the preceding 3 months. The exclusion criteria were as follows: (1) inability to comply with treatment or complete scales; (2) severe insomnia and anxiety induced by organic diseases and psychiatric drugs; (3) diagnosed with severe anxiety, depression, schizophrenia, or other psychiatric conditions; (4) concurrent severe cardiac, hepatic, renal dysfunction, or other significant organic diseases; (5) breastfeeding women or pregnant women; and (6) conditions of the acupoint skin such as ulceration, swelling, or allergy rendering acupuncture unsuitable. The rejection and drop‐out criteria were as follows: (1) suboptimal adherence to medication, failing to take prescribed drugs as required; (2) failure to undergo treatment as scheduled or incomplete data compromising the assessment of efficacy and safety; (3) voluntary drop‐out or loss to follow‐up during observation; (4) combined use of treatment methods prohibited by this protocol or changing treatment plans midway on their own; and (5) physical unsuitability for continued treatment. The observation termination criteria were as follows: (1) ineligibility for further study participation due to severe adverse reactions; (2) subject requests for mid‐study withdrawal; (3) worsening of the subject's condition necessitating urgent interventions; and (4) non‐cooperation from the patient, with ineffective communication attempts by the investigator. This study adheres to the Declaration of Helsinki and was approved by the hospital's ethics committee, with the ethical approval number 2020SYKY03‐01. All patients provided signed informed consent.

### 2.2 Study Methods

Intervention measures: Acupuncture and TCM Group: Participants received acupuncture treatment while taking traditional Chinese medicine (TCM) decoctions. The TCM formulation consists of Radix Bupleuri (9 g), Tangerine Peel (9 g), Szechwan Lovage Rhizome (6 g), Rhizoma Cyperi (6 g), Fructus Aurantii (6 g), Paeonia Lactiflora (6 g), and Prepared Liquorice Root (3 g). The regimen for TCM treatment involves administration twice daily, 15–30 min post‐meal with warm water, over a period of 4 weeks, constituting a single course of treatment. Single‐use sterile acupuncture needles by the Andi brand (Guizhou Andi Medical Equipment Co. Ltd.) (0.30 × 40 mm, 0.25 × 25 mm, length 1–1.5 inches) were utilized for acupuncture. Localization followed the method outlined in the “Meridian and Acupoints (Shen 2016),” a national‐level textbook for higher education during the “Thirteenth Five‐Year Plan.” Selected acupoints are Yintang, Shenmen, Zhaohai, Shenmai, Xingjian, and Taichong. The acupuncture technique involved the patient in an appropriate position, routine disinfection of the acupoint areas by the research team's physician, employing the even reinforcing‐reducing method. After bringing about the desired Qi, needles were retained for 30 min. The acupuncture regimen was once daily, five times weekly, over 4 weeks.

TCM group: Participants received TCM decoctions. The treatment plan of TCM decoction was the same as that of Acupuncture and the TCM Group.

Control group: Patients in this group received oral administration of dexzopiclone tablets (dexzopiclone tablets, specification: 3 mg per tablet, batch number: NMPA approval No. H00074, produced by Chengdu Kanghong Pharmaceutical Group Co. Ltd.), once daily at bedtime, over a 4‐week course.

Compliance evaluation: Patient treatment compliance = number of treatments received by the subject/total number of treatments the subject was supposed to receive × 100%.

### 2.3 Data Collection

Data collected included general patient information, PSQI and TCM constitution scores before treatment and one month post‐treatment, as well as HAMA and HAMD scores before treatment, after 4 weeks, and after 8 weeks of treatment, along with treatment efficacy rates and safety indicators during treatment. General information included gender, age, BMI, and duration of illness.

Sleep quality was assessed using the Pittsburgh Sleep Quality Index (PSQI) (Yang et al. 2023), including seven domains: subjective sleep quality, sleep latency, sleep duration, habitual sleep efficiency, sleep disturbances, use of sleep medication, and daytime dysfunction. The total score, the sum of scores across these domains, ranges from 0 to 21, with higher scores indicating worse sleep quality. Scores are categorized as follows: 0–5 indicates very good sleep quality; 6–10, fairly good sleep quality; 11–15, average sleep quality; 16–21, very poor sleep quality.

The Hamilton Anxiety Scale (HAMA) evaluates anxiety symptoms before and after care; scores < 7 indicate no anxiety symptoms and psychological normality; scores > 7 but < 14 suggest possible anxiety symptoms; scores ≥ 14 but < 21 confirm anxiety symptoms; scores ≥ 21 but < 29 indicate significant anxiety symptoms; scores ≥ 29 denote severe anxiety symptoms. The score is inversely proportional to the severity of anxiety symptoms, that is, the lower the score, the lighter the anxiety symptoms (Ao et al. 2024).

The HAMD, created by Hamilton in 1960, features 24 items across 7 categories: anxiety/somatization, weight, cognitive impairment, diurnal variation, retardation, sleep disturbances, and feelings of despair. Outcome criteria are as follows: a total score > 35 indicates severe depression, 21–35 indicates mild to moderate depression, 8–20 indicates borderline depression, and < 8 indicates no depression (Lee Duckworth et al. 2005).

Clinical comprehensive efficacy was assessed according to criteria from the “Guidelines for Clinical Research of New Chinese Medicines in the Treatment of Insomnia”: Cured: defined as normalization of sleep maintenance time or nighttime sleep exceeding 6 h, with deep sleep and refreshed spirit upon waking. Markedly effective: defined as significant sleep quality improvement, with sleep time increasing by more than 3 h and deeper sleep onset. Effective: defined as alleviated insomnia symptoms, with sleep time increasing by less than 3 h compared to before. Ineffective: defined as no significant change or worsening of insomnia symptoms post‐treatment. The total effective rate = (number of clinically cured cases + number of markedly effective cases + number of effective cases) / total number of cases × 100%.

Safety indicators included routine blood tests, routine urine tests, liver function, and kidney function.

### 2.4 Statistical Analysis

All data were statistically analyzed using the SPSS 26.0 software (SPSS Inc., Chicago, IL, USA). The normality of data was assessed through the K–S test. Quantitative data that followed a normal distribution were expressed as mean ± standard deviation (x̄ ± s). For comparisons of mean values across multiple groups, one‐way analysis of variance (ANOVA) was utilized, with subsequent pairwise comparisons made using the SNK method. For repeated measures data, repeated measures ANOVA was applied. Categorical data were presented as frequencies (*n*) or percentages (%), and analyzed using the chi‐square (*χ* ^2^) test. A *p*‐value < 0.05 was considered a statistically significant difference.

## 3 Results

### 3.1 General Data

Initially, 136 patients were enrolled in this study, of which 120 completed the entire treatment process. The analysis was based on data from these 120 patients. All patients managed to complete the treatment protocol within the designated timeframe, effectively cooperated with the medical staff in completing assessment scales, and demonstrated satisfactory compliance. The findings revealed that the acupuncture and TCM group comprised 40 patients (25 males and 15 females) with an average duration of illness of 15.70 ± 5.40 months; the TCM group also included 40 patients (29 males and 11 females) with an average illness duration of 16.80 ± 5.60 months; and the control group consisted of 40 patients (28 males and 12 females) with an average disease duration of 15.43 ± 4.33 months. No significant differences were observed among the groups regarding gender, age, BMI, duration of illness, and comorbid disease(*p* > 0.05), as detailed in Table 1.

**TABLE 1:** Population characteristics of the three groups.

Item	Acupuncture group (*n* = 40)	Chinese medicine group (*n* = 40)	Control group (*n* = 40)	*p*
Sex (male/female)	25/15	29/11	28/12	0.606
Age (years)	45.19 ± 10.60	42.80 ± 8.70	43.57 ± 9.45	0.377
BMI (kg/m^2^)	23.48 ± 2.11	23.56 ± 2.02	23.49 ± 2.12	0.524
Course (months)	15.70 ± 5.40	16.80 ± 5.60	15.43 ± 4.33	0.415
Hypertension	4	3	6	0.218
Diabetes	7	6	4	0.363
Chronic pain	2	4	3	0.439

### 3.2 Comparison of Sleep Quality

Before treatment, no significant differences were found among the three groups in sleep quality, sleep onset time, sleep duration, sleep efficiency, sleep disturbances, daytime dysfunction, and overall scores (*p* > 0.05). Post‐treatment, all three groups exhibited improvements in sleep quality, sleep onset time, sleep duration, sleep efficiency, sleep disturbances, daytime dysfunction, and overall scores compared with before treatment (*p* < 0.05). Statistically significant differences were observed among the three groups in these indicators post‐treatment (*p* < 0.05). Further pairwise comparisons indicated that, regarding sleep quality, sleep onset time, daytime dysfunction, and overall scores, the ranking of groups was as follows: acupuncture and TCM group > TCM group = control group; for sleep duration, sleep efficiency, and sleep disturbances, the ranking was: acupuncture and TCM group > TCM group > control group, as detailed in Table 2.

**TABLE 2:** Sleep quality of the three groups.

Item	Time	Acupuncture group (*n* = 40)	Chinese medicine group (*n* = 40)	Control group (*n* = 40)	*p*
Sleep quality	Before treatment	2.11 ± 0.81	2.43 ± 0.66	2.60 ± 0.50	0.065
	After treatment	1.52 ± 0.55^a^	1.70 ± 0.78^a^	1.67 ± 0.18^a^	<0.001
Time before falling asleep	Before treatment	2.16 ± 0.68	2.20 ± 0.63	2.83 ± 0.46	0.780
	After treatment	1.34 ± 0.61^a^	1.70 ± 0.77^a^	1.74 ± 0.55^a^	<0.001
Sleep time	Before treatment	2.66 ± 0.75	2.25 ± 0.65	2.83 ± 0.46	0.610
	After treatment	1.48 ± 0.55^a^	1.73 ± 0.72^a^	2.01 ± 0.39^a^	<0.001
Sleep efficiency	Before treatment	2.95 ± 0.71	2.46 ± 0.71	2.67 ± 0.71	0.180
	After treatment	1.34 ± 0.48^a^	1.73 ± 0.77^a^	2.03 ± 0.32^a^	<0.001
Sleep disorders	Before treatment	2.39 ± 0.75	2.30 ± 0.67	2.57 ± 0.68	0.405
	After treatment	1.20 ± 0.41^a^	1.73 ± 0.45^a^	1.92 ± 0.44^a^	<0.001
Daytime dysfunction	Before treatment	2.73 ± 0.74	2.41 ± 0.69	2.50 ± 0.51	0.234
	After treatment	1.23 ± 0.74^a^	1.90 ± 0.51^a^	2.11 ± 0.45^a^	<0.001
Total score	Before treatment	15.00 ± 1.92	15.75 ± 1.74	16.00 ± 1.39	0.848
	After treatment	8.11 ± 1.17^a^	10.60 ± 1.48^a^	10.87 ± 1.01^a^	<0.001

^a^Compared with before treatment, the difference was statistically significant.

### 3.3 Comparison of Anxiety and Depression Scores During Treatment

Utilizing one‐way repeated measures ANOVA, the study explored the effects of various treatment approaches on the HAMA and HAMD scores of patients within an 8‐week timeframe. The Shapiro–Wilk test indicated that the data across all groups adhered to an approximate normal distribution (*p* > 0.05), and Mauchly's test of sphericity verified the equality of the covariance matrices across the groups (*p* > 0.05). The data were expressed as mean ± standard deviation (x̄ ± s), revealing no significant differences in the initial HAMA and HAMD scores among the groups (all *p* > 0.05), indicating a high level of comparability, as depicted in Table 3. A summary of the findings is as follows:

**TABLE 3:** Levels of anxiety and depression in the three groups.

Item	Time	Acupuncture group (*n* = 40)	Chinese medicine group (*n* = 40)	Control group (*n* = 40)	*p*_interaction_	*p*_time_	*p*_treatment_
HAMA	Baseline	12.53 ± 3.47	11.97 ± 1.43	12.17 ± 1.65	0.122	< 0.001	0.173
	4 weeks	8.07 ± 2.12^a^	7.47 ± 2.74^a^	7.55 ± 2.14^a^			
	8 weeks	7.63 ± 2.56^a^	7.60 ± 2.72^a^	7.41 ± 1.74^a^			
HAMD	Baseline	6.07 ± 1.17	5.80 ± 0.40	6.02 ± 0.45	0.283	< 0.001	0.145
	4 weeks	5.07 ± 1.28^a^	4.90 ± 1.53^a^	5.13 ± 1.04^a^			
	8 weeks	4.70 ± 1.51^a^	5.10 ± 1.94^a^	4.97 ± 1.23^a^			

^a^Compared with baseline, the difference was statistically significant.

There was no significant interaction between time and treatment for HAMA and HAMD scores (all *p* > 0.05), indicating that the magnitude of the individual effects of different treatment modalities on HAMA and HAMD scores was consistent across the three groups at the three assessed time points. Moreover, there was a reduction in HAMA and HAMD scores over time across all groups (all *p* < 0.001), signifying a notable temporal change in HAMA and HAMD scores.

Ultimately, the impact of the various treatment modalities on HAMA and HAMD scores was consistent across all groups (all *p* > 0.05). Comparisons with baseline data revealed that the HAMA and HAMD scores at weeks 4 and 8 were significantly lower than those at the baseline (*p* < 0.05).

This study did not compare the groups of individuals with depression and anxiety, so it is not possible to conclude any differences in therapeutic efficacy between depression and anxiety.

### 3.4 Comparison of Constitution Improvement

Prior to treatment, there were no significant differences in the constitution scores among the three groups (*p* > 0.05). Post‐treatment, both the acupuncture and medication group and the medication group exhibited reductions in constitution scores (*p* < 0.05). The greater the decrease in constitution scores, the better the therapeutic effect of improving constitution. A significant variance in constitution scores was observed post‐treatment among the groups, with further pairwise comparisons indicating the scores in descending order as: acupuncture and TCM group > TCM group > control group, as detailed in Table 4.

**TABLE 4:** The physical improvement of the three groups before and after treatment.

Time	Acupuncture group (*n* = 40)	Chinese medicine group (*n* = 40)	Control group (*n* = 40)	
Before treatment	78.47 ± 10.08	79.87 ± 9.15	79.21 ± 8.23	0.247
After treatment	61.50 ± 12.83^a^	68.97 ± 13.78^a^	77.59 ± 9.65	< 0.001

^a^Compared with before treatment, the difference was statistically significant.

### 3.5 Comparison of Clinical Efficacy

**FIGURE 2:**
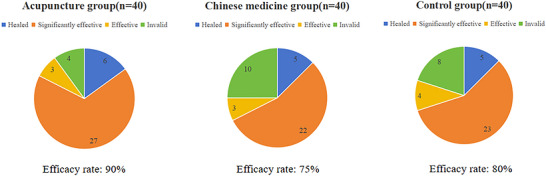
Comparison of clinical efficacy.

The efficacy outcomes revealed that within the acupuncture and TCM group, 36 cases were effective and 4 were ineffective, resulting in an efficacy rate of 90%; within the TCM group, 30 cases were effective and 10 were ineffective, leading to an efficacy rate of 75%; within the control group, 32 cases were effective and 8 were ineffective, with an efficacy rate of 80%. The variance in efficacy rates among the groups did not reach statistical significance (*p* = 0.210), as detailed in Table 5. For the efficacy rate, the ranking was: acupuncture and TCM group > control group >TCM group, as shown in Figure 2.

**TABLE 5:** The clinical efficacy of three groups.

Item	Healed	Significantly effective	Effective	Invalid	Effective rate (%)
Acupuncture group (*n* = 40)	6	27	3	4	90
Chinese medicine group (*n* = 40)	5	22	3	10	75
Control group (*n* = 40)	5	23	4	8	80
*p*	0.210				

### 3.6 Observations on Safety

Throughout this study involving 120 patients, all acupuncture procedures were meticulously executed by qualified physicians from the acupuncture department, adhering to stringent standards without any clinical adverse incidents such as syncope, needle breakage, retained needles, or bruising. During the course of oral medication treatment, there were no reported adverse reactions such as nausea, vomiting, liver impairment, or renal impairment.

## 4 Discussion

The doctrine of TCM asserts that constitution is an objective life phenomenon intricately linked to the genesis, evolution, and resolution of diseases (Q. Wang 2008). Qi stagnation constitution delineates a relatively stable trait of the human body, in contrast to Liver Qi stagnation syndrome, which is more vulnerable to external factors. The Qi stagnation constitution emphasizes the individual, whereas Liver Qi stagnation syndrome is disease‐centric. Predominantly characterized by depressive moods and anxiety, a prolonged Qi stagnation constitution can lead to Liver Qi discomfort and impeded heart blood circulation, eventually culminating in insomnia due to the inability of Yang to merge with Yin. Individuals with a Qi stagnation constitution often exhibit psychological issues of varying severity, with sleep and internal psychology influencing each other. Chronic difficulties in initiating sleep can provoke irritability and predispose individuals to depression, anxiety, and similar disorders. Conversely, conditions such as anxiety and depression can deteriorate sleep quality, perpetuating a cyclical pattern. In an investigation into insomnia's risk factors, Zhao and Jia (2015) analyzed 500 insomnia patients, identifying a predominant presence of Qi stagnation constitution. X. C. Jiang et al.'s (2020) survey on the constitution of 160 insomnia patients revealed a predominance of imbalanced constitutions, mainly Qi stagnation, Qi deficiency, and Yang deficiency, all of which impact sleep quality.

The formulation employed in this study, designed to facilitate Qi circulation and alleviate Qi stagnation, comprises seven Chinese medicinal herbs. Contemporary pharmacological investigations have revealed that saikosaponin possesses significant target‐binding efficacy, potentially underpinning the formula's therapeutic action against insomnia (Liu et al. 2019); Radix Paeoniae Alba's primary chemical, paeoniflorin, notably enhances the secretion of endogenous substances within the cerebrospinal fluid, thus ameliorating sleep (Y. F. Li et al. 2014). Reports suggest that Rhizoma Cyperi's volatile oil significantly affects the efficacy of nitrazepam and benzodiazepines through its transdermal enhancement effect (Zhou 2012). This volatile oil can ameliorate depressive symptoms in mice by modulating central cholinergic systems and elevating serotonin levels (S. Y. Li and Xie 2018). Fructus Aurantii extract exhibits antidepressant capabilities (Yi et al. 2010). Prepared Liquorice Root, rich in *Glycyrrhiza glabra* L. and glycyrrhizin, is frequently utilized in depression treatments (Y. L. Zhang et al. 2019). *Glycyrrhiza glabra* L. extracts can prevent and treat depression by inhibiting cortisol elevation and modulating the HPA axis (H. F. Li 2008), indicating the formula's efficacy in addressing insomnia associated with Qi stagnation factors. The formula employs Radix Bupleuri among other herbs to disperse liver Qi and relieve depression, thereby optimizing liver function and normalizing Qi flow. Concurrently, it uses *Paeonia lactiflora* and similar herbs to enrich the blood and soften the liver, fostering nourishment for the liver and harmonious operation of organ functions, thus mitigating the imbalanced Qi stagnation constitution state (Ma and Zhu 2013).

Acupuncture, a prevalent TCM clinical approach for managing insomnia, anxiety, and other mental disorders, offers bidirectional regulatory functions and significant efficacy without the adverse effects associated with sedative‐hypnotic drugs. The principal theory behind acupuncture treatment for insomnia is that it facilitates internal‐external communication, supplements deficiencies, drains excess, regulates the organs, supports the righteous, and expels the pathogenic, thereby improving sleep quality. Acupuncture can effectively ameliorate insomnia symptoms, potentially related to the regulation of certain neurotransmitters and brain regions. It can not only regulate the content of GABA and GABAA receptors (Chinese Association of Traditional Chinese Medicine 2009) but also adjust the levels of monoamine neurotransmitters 5‐HT, 5‐HIAA, NE, and cytokine IL‐1β in the brain (X. M. Zhang 2008), thereby improving sleep and anxiety. Research indicates (J. C. Lu and Zhang 2002) that sleep‐related factors such as adenosine (AD), melatonin (MT), prostaglandin (PG), interleukins (IL), and tumor necrosis factor (TNF) play crucial roles in sleep mechanisms. Acupuncture regulates sleep by adjusting these sleep factors. Other modern studies (J. Lu et al. 2000) have also found that stimulating related acupoints on the head can activate the cerebral cortex and sleep center, smoothing different neural pathways, easing emotions, relieving stress, promoting sleep, and improving sleep quality.

The current analysis results show that acupuncture combined with Chinese medicine seems to have a more positive effect than Western medicine alone in improving sleep in Perimenopausal insomnia patients (Z. Li, Yin, et al. 2023). This was consistent with the results of this study. The findings of this study indicate that the combined treatment of TCM and acupuncture for insomnia associated with Qi stagnation constitution is superior in reducing subjective sleep quality, sleep duration, habitual sleep efficiency, daytime dysfunction, total PSQI score, and TCM Qi stagnation constitution score compared to the use of TCM alone and conventional Western medicine. The potential reasons are as follows: on one hand, the efficacy of the TCM group in this study is to soothe the liver, regulate Qi, relieve depression, and calm the mind, while the acupuncture group's selection of acupoints and needling techniques also have the effects of soothing the liver and calming the heart, calming the mind, and aiding sleep, thus the therapeutic effects are cumulative and enhanced; on the other hand, the treatment sites for acupuncture are mainly meridians, which balance Yin and Yang by regulating the Qi of the meridians, offering rapid efficacy and constituting an external treatment, whereas medication treatment is an internal treatment, and their combination broadens the treatment scope.

Nonetheless, the study is not without its limitations. The small sample size and brief observation period diminish the representativeness of the findings for the broader population of patients suffering from Qi stagnation constitution‐related insomnia; the absence of long‐term follow‐up precludes a comprehensive understanding of the enduring effects of the treatment; the reliance on subjective rather than objective measures weakens the empirical foundation of the results; and the study's focus on constitutional aspects without integrating TCM syndrome differentiation represents a significant oversight, leaving a gap in the holistic understanding of treating constitution and syndrome in tandem. It is hoped that future opportunities will allow for the improvement of the aforementioned deficiencies and a deeper study into insomnia.

## 5 Conclusion

The efficacy of treating Qi stagnation type insomnia with a combination of TCM and acupuncture is validated, offering significant relief from clinical symptoms of insomnia(90% efficacy rate). The combination of TCM and acupuncture can improve sleep quality, alleviate anxiety and depression, improve physical fitness, and has a high clinical efficacy. Compared to using TCM decoction alone for treatment, the combination of TCM decoction and acupuncture has more advantages (90% efficacy rate vs. 75% efficacy rate).

## Author Contributions

**Peng Bai**: conceptualization, writing – review and editing. **Xue‐Wen Mao**: writing – original draft, data curation, formal analysis.

## Funding

This study was funded by Young Qihuang Scholars Cultivation Project, project number: National Traditional Chinese Medicine Education Letter [2022] 256; National Traditional Chinese Medicine Innovation Backbone Talent Project, project number: National Traditional Chinese Medicine Education Letter [2019] 128; Capital Health Development Research Program, project number: 2024‐2‐7104.

## Ethics Statement

The study followed the basic principles of the Declaration of Helsinki and was approved by the Ethics Committee of Shunyi Hospital, Beijing Traditional Chinese Medicine Hospital (2020SYKY03‐01). Written informed consent was obtained from all participants.

## Conflicts of Interest

The authors declare no conflicts of interest.

## Data Availability

All data generated or analyzed during this study are included in this published article.
